# Inhibition of regulated cell death by cell-penetrating peptides

**DOI:** 10.1007/s00018-016-2200-7

**Published:** 2016-04-05

**Authors:** Stefan Krautwald, Christin Dewitz, Fred Fändrich, Ulrich Kunzendorf

**Affiliations:** 1 Department of Nephrology and Hypertension, University Hospital Schleswig-Holstein, 24105 Kiel, Germany; 2 Clinic for Applied Cellular Medicine, University Hospital Schleswig-Holstein, 24105 Kiel, Germany

**Keywords:** Cell-penetrating peptide (CPP), Protein transduction domain (PTD), Regulated cell death (RCD), Protein therapy

## Abstract

Development of the means to efficiently and continuously renew missing and non-functional proteins in diseased cells remains a major goal in modern molecular medicine. While gene therapy has the potential to achieve this, substantial obstacles must be overcome before clinical application can be considered. A promising alternative approach is the direct delivery of non-permeant active biomolecules, such as oligonucleotides, peptides and proteins, to the affected cells with the purpose of ameliorating an advanced disease process. In addition to receptor-mediated endocytosis, cell-penetrating peptides are widely used as vectors for rapid translocation of conjugated molecules across cell membranes into intracellular compartments and the delivery of these therapeutic molecules is generally referred to as novel prospective protein therapy. As a broad coverage of the enormous amount of published data in this field is unrewarding, this review will provide a brief, focused overview of the technology and a summary of recent studies of the most commonly used protein transduction domains and their potential as therapeutic agents for the treatment of cellular damage and the prevention of regulated cell death.

## Cell-penetrating peptides and their composition

The hydrophobic nature of cellular membranes and the blood–brain barrier selectively restrict cells from taking up exogenous molecules larger than 500 Da. This is one reason why virus-mediated gene expression was considered for a long time as the most efficient and reliable approach for expressing bioactive proteins de novo, aiming to correct defects of proteins involved in a plethora of different genetic-metabolic disorders and diseases. Nevertheless, viral vectors are capable of integrating into the host chromatin and this may have serious consequences from long-term effects which limit the clinical and therapeutic applications of virus-associated material such as adenoviruses, herpes viruses or lentiviruses [1]. An impressive alternative strategy to penetrate the impermeable phospholipid bilayer of cell membranes and to cross biological barriers is derived from the HIV transactivator (Tat) protein. Following secretion from HIV-infected cells, Tat translocates into neighboring cells to modify gene transcription and spread the disease [2]. The first report that demonstrated that a Tat-derived peptide can deliver a large protein into different cell types and mammalian organs was published by Fawell and colleagues in 1994 [3]. Initially, they chemically cross-linked and identified a 36-amino acid region of HIV-1 that was able to promote the uptake of β-galactosidase as a chimera into living cells. This HIV Tat protein transduction domain (PTD) contains a cluster of basic amino acid residues and a sequence assumed to adopt an α-helical configuration. Countless studies aimed to delineate whether shorter domains of this Tat peptide would be sufficient for cell internalization. The main determinant required for translocation was identified as the cluster of basic amino acids, while the putative α-helix domain appeared dispensable, although peptides with an α-helical region can more efficiently enter cells. The research group led by Bernard Lebleu finally identified the truncated polycationic peptide GRKKRRQRRR that includes RNA binding and nuclear localization signal (NLS) motifs which was adequate for effective translocation into cells and tissues [4]. At almost the same time, the group of Gilles Divita designed, with a short peptide vector termed MPG, the first non-covalent cell-penetrating peptide (CPP) for delivery of nucleic acids into cultured cells [5] closely followed by the development of Pep-1, a peptide carrier for the cellular transfer of peptides and proteins [6]. Complementary studies suggest that at least eight positive charges are required for efficient penetration by individual CPPs [7]. Remarkably, too few positively charged amino acids of the CPPs or PTDs results in poor cell adsorption and low cellular delivery, respectively, while too many charges leads to toxicity [8]. Nevertheless, commonly used CPPs are passive carriers that do not initiate release of damage-associated molecular patterns (DAMPs) or inflammatory cytokines during the process of cytoplasmic delivery of the cargo [9]. The aforementioned decapeptide (GRKKRRQRRR) is the prototypic CPP whereas in general, CPPs are relatively short peptides (normally ≤40 amino acids) classified into cationic, amphipathic and hydrophobic types according to their physical–chemical properties. CPPs are able to enter cells even across the blood–brain barrier by passive, temperature-, energy- and receptor-independent processes and deliver otherwise non-permeable cargos like proteins in a bioactive, nontoxic manner [10]. The course and dynamics result in immediate bioavailability of the cargo. Although such routes may still be valid for the most part, recent observations have demonstrated that so-called Xentry (LCLRPVG), which derived from an N-terminal region of the X-protein of the hepatitis B virus, represents a new class of CPPs that enters cells exclusively via an energy-dependent endocytotic process, thereby extending the range of pathways of cellular uptake [11]. Similarly, it is worth mentioning that recent studies suggest that a receptor-mediated uptake cannot be ruled out for any kind of CPP-conjugate [12]. Studies and exploitation of CPPs now extend into a third exciting decade, and since the initial discovery of Tat-mediated delivery, many other promising transduction domains have been discovered. Meanwhile, hundreds of sequences now fall within the cell penetrating peptide classification. The most widely used CPPs are listed in Table 1.

**Table 1:** Commonly used cell-penetrating peptides (CPPs)

Peptide	Sequence	References
HIV-1 Tat	GRKKRRQRRR	[123]
MAP	KLALKLALKALKAALKLA	[124]
MTS	AAVALLPAVLLALLAP	[125]
MPG	GLAFLGFLGAAGSTMGAWSQPKKKRKV	[5]
Penetratin^a^	RQIKIWFQNRRMKWKK	[23]
Pep-1	KETWWETWWTEWSQPKKRKV	[6]
Poly-arginine (R8-R12)	RRRRRRRR-RRRRRRRRRRRR	[126]
Transportan	GWTLNSAGYLLGKINLKALAALAKKIL	[127]
VP22	DAATATRGRSAASRPTQRPRAPARSASRPRRPVQ	[128]

^a^Derived from the *Drosophila* Antennapedia homeodomain (residues 43–58)

Although there seems to be little or no homology between the primary and secondary structures of the different PTDs, the efficacy of cellular transduction has been found to correlate strongly with the number of basic amino acids. Although there is no restriction on the size or type of the delivered cargo, the capacity for successful uptake of cargoes into cells increases when the cationic PTD is attached to lower molecular weight compounds [13, 14]. CPPs enter cells by various mechanisms including direct translocation through the membrane and clathrin-independent macropinocytosis but the exact pathway of cellular uptake has not been entirely resolved [15]. Likewise, it is still not clear if there is a correlation between the peptide secondary structure and its ability to transduce into cells. That the transduction efficacy of proteins with PTD or CPPs, mediated by the positively charged arginine and lysine residues within the peptide, could be abolished by the addition of highly negatively charged molecules strongly suggests that the transduction process occurs in a manner dependent on the presence of sialic acid residues present in glycosphingolipids or heparin sulfate proteoglycans that are expressed ubiquitously on the cell surface [16]. However, using cells deficient in glycosaminoglycans and sialic acids, the group of Steven Dowdy has demonstrated that PTD-mediated induction of macropinocytosis and cellular transduction of CPPs occurs just as efficiently in the absence of heparin sulfate and sialic acid [17]. It has become clear, however, that, at least for many cationic CPPs, binding to glycosaminoglycan is a substantial step before transduction into the cell, but above a concentration threshold (generally in the low micromolar range) CPPs can also penetrate the membrane directly [18]. In general, the individual nonhomogeneous composition, density and fluidity of the lipid bilayer of various cell types affect the rate and mode of uptake of CPPs [19]. Peptides with the correct physicochemical composition might be able to cross the cell membrane directly and would be immediately available in the cytosol, closely resembling the behavior of small molecules [20]. In this context, it is worth mentioning that the Tat-domain alone, without any further cargo, possesses intrinsic neuroprotective properties in vitro as well as in vivo [21] and simple poly-arginine (up to R18) has high neuroprotective potency in stroke models relative to other PTDs or CPPs [22]. The cytoprotective properties of poly-arginine, probably mediated by interfering with NMDA signaling, highlight the need to interpret the abundant neuroprotection studies using CPPs as delivery agents with caution, but indicates that they are ideal carrier molecules to deliver neuroprotective drugs to the CNS following injury like cerebral ischemia, Parkinson’s disease or Alzheimer’s disease. In addition, remarkable is the observation that cationic CPPs themselves (without further cargo) are occasionally able to downregulate TNF receptors at the cell surface which could inhibit TNF-mediated signal transduction in this setting.

## Final location of CPPs after cellular entry

**Fig. 1:**
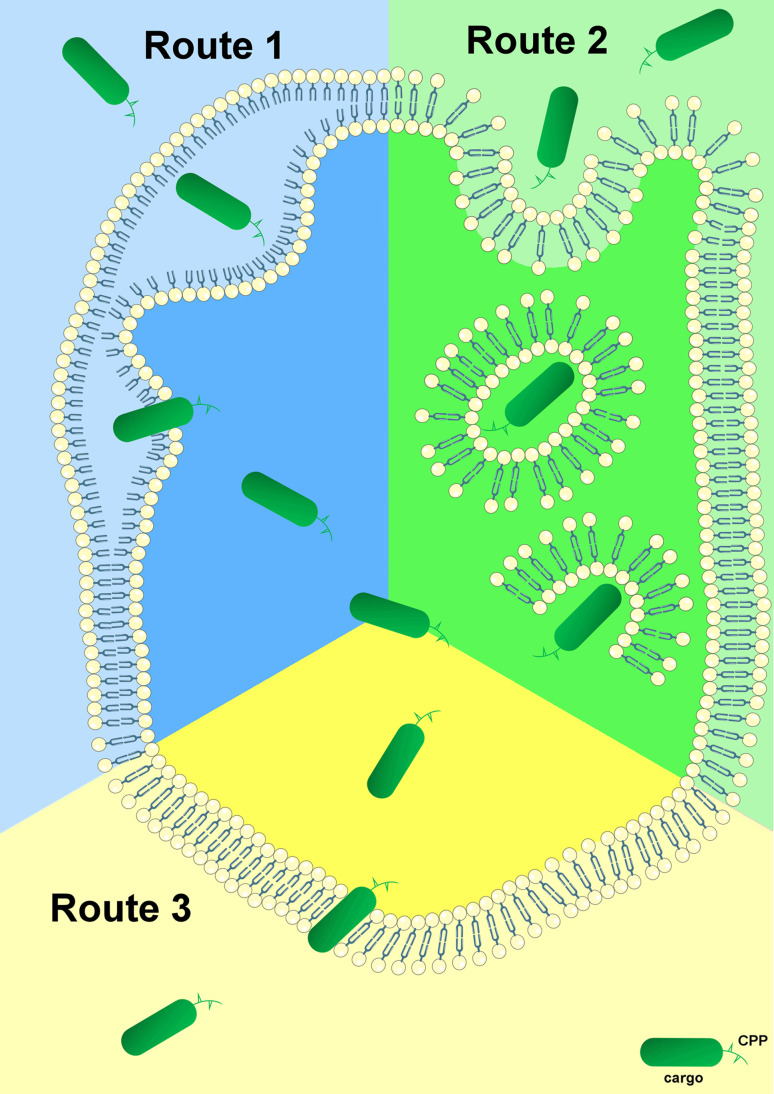
Proposed mechanisms for cellular internalization of CPPs. First of all, each CPP-conjugate binds to the plasma membrane via electrostatic interactions. Subsequently, the complete conjugate is internalized and released through various conceivable mechanisms. *Route 1* represents cell entry of the CPP-complex through the formation of an inverted micelle (aggregates of colloidal surfactants in which the polar groups are concentrated in the interior and the lipophilic groups extend outward into the solvent). The majority of CPPs probably enter cells by an endocytosis-driven pathway which is depicted as *Route 2*. *Route 3* is a direct, energy- and receptor-independent penetration and transduction process of the construct through the plasma membrane

To elucidate the exact mechanisms of cellular entry, CPPs have been intensively studied for the last two decades. The most proposed feasibilities of cellular delivery of cargoes mediated by CPPs are illustrated in Fig. 1. In contrast to these mechanisms of cell entry for a plethora of CPPs, little is known about the subsequent intracellular cytosolic trafficking of the penetrated molecules which is of course crucial for the cargo to reach its intended target. Once inside the cell, CPP-linked cargoes remain in the cytoplasm or move to other compartments whereas specific targeting to particular organelles requires the addition of extra “address motifs” within the PTD sequence. Therefore, it is possible to alter the distribution of a cargo by modifying the CPP itself. For example, mutation of three amino acids within the penetratin sequence maintains it within the cytoplasm instead of its default localization in the nucleus [23]. Furthermore, using a variety of subcellular localization sequences, organelle-specific directed delivery of CPPs to the endoplasmic reticulum and mitochondria has been described in mammals [24, 25]. The enhancement of cell specificity using activatable cell-penetrating peptides (ACPPs) [26] and the aforementioned targeted transport of cargo into specific organelles by insertion of corresponding localization sequences are only two promising developments for the purpose of specific therapeutic applications of CPP-conjugates. ACPPs consist of a polycationic domain connected via a linker to a neutralizing polyanion which reduces the overall charge to nearly zero and thus inhibits electrostatic uptake into cells. The linker can be cleaved by enzymes produced in cancer cells (preferably matrix metalloproteinase-2 and -9). This activates the cell-penetrating properties of the peptide, allowing specific entry into cancer cells [27]. This *modus operandi* may be a novel chemotherapeutic approach that specifically targets the activity of CPP-conjugated anticancer drugs to tumor tissue.

Beyond this, the nature of the link between the vector (CPP or PTD) and the cargo is also of importance. If these two elements are linked through a disulfide bridge, the cargo is rapidly released through the action of cytoplasmic glutathione. If the link is permanent, as in a fusion protein, then the final localization of the chimeric molecules will depend on the properties of both elements [28]. Nevertheless, the major rate-limiting step of CPP-mediated drug delivery is the escape of the cargo from endosomes into the cytoplasm and/or nucleus of the target cell, whereas only a small fraction of CPPs fulfill these requirements [29]. Therefore, extensive and also individual experimentation is required and of paramount importance to determine the optimal CPP for any given cargo and cell type to escape this bottleneck. The most promising efforts towards enhancing endosomal escape without increasing cell toxicity take their cue from mechanisms that are used by enveloped viruses like influenza or retro-viruses to escape endosomes during infection [30]. To discover translocated molecules, different methods for detecting the stability and activity of CPPs and cargoes in relation to proteolytic cleavage have been widely developed [31]. For instance, the amount and proteolytic cleavage of internalized CPP-conjugates is often studied by HPLC with fluorescent detection and by MALDI-TOF MS analysis [32].

Another sophisticated approach used a transducible Tat-Cre recombinase reporter assay in viable cells where the functionality of the transduced cargo was indicated and confirmed through the genetic reconstitution of EGFP expression [10]. Such proceedings are extremely convenient for the purpose of designated therapeutic applications of the cargo.

Pharmacokinetic studies of the commonly used CPPs (listed in Table 1) proved that the distribution of radiolabeled conjugates in mammals in general showed a high transient accumulation of the injected drugs in well-perfused organs and rapid clearance from circulation. All of the tested CPPs revealed a relatively low accumulation rate of the peptides in the brain, whereas the highest uptake values were detected in the liver and the kidneys [33]. The data herein support individual design of peptide-based therapeutics, particularly for topical application.

## Regulated cell death (RCD) as innovative scope of CPP application

Retrospectively, the evidence that large Tat-domain-conjugated enzymes are indeed able to transduce mammalian cells in vivo and that these proteins can be delivered with high efficiency and preserved biological function in a whole organism was reported for the first time in 1999 [34]. In the aftermath of this landmark paper, many manuscripts were published on this topic, including many false-positive artificial interpretations and reports on cells that were fixed after treatment with dye-labeled CPPs [35]. In general, the main difficulty consists in correctly judging whether a substance is just bound to the cell surface or taken up into the cell. This is a challenge, but simple western-blots are often sufficient to definitively answer the question.

Meanwhile, there are a plethora of different variants of CPPs and PTDs described in the literature [36]. The number of applications using peptide carriers for the delivery of therapeutically relevant molecules is continuously increasing and so far more than 300 studies from in vitro to in vivo have been reported [13]. From a clinical perspective, effective delivery of recombinant proteins might result in the therapeutic replacement of dysfunctional or missing proteins. Of course, due to the comparatively short circulating half-life in vivo, these recombinant drugs often need to be administered frequently. Uptake of functional, biologically active recombinant proteins offers a promising opportunity to agonize or antagonize signal transduction pathways which are mediated by or involved in a variety of molecularly controlled processes resulting from regulated cell death (RCD) and culminating in tissue injury and inflammation. RCD is a physiological process that controls homeostatic events whose deregulation can lead to the development of a number of human diseases and tissue damage [37]. Apoptosis is the most studied and best described form of caspase-dependent regulated cell death, but current investigations indicate that RCD can also occur via regulated necrotic pathways. At the moment, the Nomenclature Committee on Cell Death has classified RCD into different subtypes in an attempt to include all available published data [38]. In this issue many outstanding experts in the field present recent insights into proteins and complex signaling mechanisms that control diverse forms of RCD including (but not limited to) necroptosis, mitochondrial permeability transition (MPT)-dependent regulated necrosis, parthanatos, ferroptosis and mitophagy. Therefore, we will refrain from listing all these effectors and pathways in detail here, and relegate at this point to the excellent reviews in this collective edition (or reviewed in [39]). A trendsetting area of CPP application includes diseases that result from RCD processes. Clinically relevant examples of this cell death form include disorders with acute or chronic courses like ischemia reperfusion injuries [40, 41], brain trauma [42], myocardial infarction [43], inflammatory diseases [44, 45], sepsis [46], oxidative stress-related disorders [47], neurodegenerative diseases [45], transplantation [48] and cancer [49], in addition to a multiplicity of other pathophysiological settings.

Furthermore, the technology and application of CPPs have contributed to the detection of a completely new form of regulated cell death which is distinct from apoptosis or necrosis. Using Tat-Beclin 1 as a cell-penetrating, autophagy-inducing synthetic peptide, the group of Beth Levine defined autosis, a novel autophagy-dependent form of cell death which is inhibitable in vitro and in vivo by cardiac glycosides [50]. Nevertheless, proof of specificity is missing in this study; above all, the scrambled Tat-Beclin 1 used therein is not the proper control peptide. To generate an optimal control peptide, essential amino acid residues must be mutated while leaving the remaining peptide sequence intact to exclude artificial effects of the penetrating construct. Furthermore, so far, it is unclear why Tat-Beclin 1 promotes autophagic cell death and whether this mode of cell death indeed represents a physiological event; understanding both would be essential to define a new type of RCD. Therefore, the enthusiasm of the scientific community regarding the existence of autosis as another form of RCD has so far remained limited [51].

## Synthesis of molecules that enable cellular uptake

To date, over 100 CPP-conjugated proteins with different functions have been transported effectively into cells in various animal models. In contrast to the fact that this technology for intracellular delivery has been applied worldwide for over 15 years, the number of proteins making it to clinical trials is manageable. Definitely, there is no lack of creative concepts. The greatest hurdle based on our own experience is not the delivery, at least in vitro, but the successful purification of recombinant proteins in a biologically active form. In the last decade, we have cloned more than 25 different fusion proteins conjugated with diverse CPPs. All of them interact with different signal transduction pathways involved in RCD, but approaches with only two of them, namely Tat-FLIP and Tat-crmA, led to exploitable findings [52, 53].

All of the different proteins could be expressed successfully in our laboratory, sometimes only after testing many different expression strains. However, an insuperable obstacle is the solubility of the recombinant proteins because most of them are preferentially enriched in bacterial inclusion bodies and this phenomenon is not dependent on the molecular size or natural occurrence of the protein in vivo. “Optimization of culture conditions” is the standard term in each troubleshooting protocol to avoid this waste of time. Of course, it is normally easy to purify a recombinant fusion protein in great quantities and qualities under denaturing conditions, but this does not solve the problem. In contrast to statements in textbooks and approved standard reports [54], we were not able to transduce even one recombinant CPP-conjugated fusion protein that was initially purified under denaturing conditions followed by a refolding process. All proteins prepared in such a manner precipitate in our hands sooner or later in vitro and certainly, *a fortiori*, in vivo. Indeed, this is the real malady of the technology, which limits the comprehensive commercialization of CPPs for clinical purposes. Of course, we are aware that our empirical experience is in contrast to many other approaches and primary publications in which denatured proteins may transduce more efficiently into cells than low energetic, correctly folded proteins, and that these denatured proteins may be correctly refolded inside the cell by chaperones [55, 56]. Obviously, these purification conditions sounded very promising not least because many recombinant proteins in a variety of expression systems lead to the formation of high level insoluble protein aggregates but unfortunately, we have not been able to reproduce this technology successfully in our lab with any recombinant fusion protein.

Using affinity tags greatly simplifies the protein purification process from crude biological extracts and thus improves the yield of the recombinant protein. If the downstream application warrants the use of a native protein, the tag must be cleaved after purification using a sequence specific protease. Of course, affinity tags may have unintended consequences, but small affinity tags like 6× His, FLAG, Strep II or CBP exert minimal effects on the structure, activity and characteristics of a recombinant protein and have limited interactions with other proteins and, therefore, usually will not need to be removed [57].

Especially for in vitro assays, the inclusion or absence of serum from the incubation medium and its influence on the capacity of CPPs to penetrate membranes is controversial [58]. At least with our commonly used conjugates we do not see any substantial differences between the presence and absence of serum. However, since most CPPs are positively charged or hydrophobic, one would assume that they bind to blood plasma proteins at least after intravenous injection in vivo, which prohibits the release of the cargo in a bioactive form after cellular uptake.

Finally, it is worth mentioning that post-translational modifications are often required for the activity of mammalian proteins and for this reason not all fusion proteins can be expressed effectively in bacteria. Currently, we know of exactly one publication that describes the generation of eukaryotic cell lines that secrete biologically active Tat fusion proteins into the culture supernatant [59].

## Therapeutic applications of cell-penetrating peptides in the context of RCD

Over the last decade, protein transduction with cell-penetrating peptides has been shown to be a highly efficient way of delivering proteins at least in vitro. Nevertheless, the in vivo application of CPPs appears to be much more complex. Often, there is a gulf between the data and results generated in a specific cell line under cell culture conditions and (patho-) physiological conditions in living mammals. Nevertheless, there are countless examples of successful applications of CPPs in treatment of human relevant injuries in the current literature, including decreased tumor growth and induction of cancer cell death (reviewed in [60]). Cargo parameters like size, structure, charge or other biophysical properties can exert a deep influence on cellular uptake and cytosolic distribution but the fact that there is no limitation on the size or type of the delivered cargo opens a wide range of opportunities illustrated by a plethora of reports [61]. Because of the main topic of this issue, we will focus in this chapter on some selected in vivo applications of CPPs and cargoes that deal with RCD, inflammation and cancer. A summary of these approaches is shown in Table 2. We do not claim that the following illustrations include all promising approaches in this field. The listed examples represent a small excerpt of the bulk of excellent in vitro studies, such as a really intriguing approach which described for the first time the incorporation of a CPP-conjugated Cas9 protein and CPP-complexed guide RNAs as a novel gene editing strategy for disrupting disease-causing genes in embryonic stem cells and other cell types [62].

**Table 2:** Selected in vivo studies using CPP-conjugated drugs as therapeutic agent of RCD

Cargo	Proposed target	Injury model	Protection	Study
FLIP	Caspase-8	Multiorgan failure in mice	Improve survival	[52]
crmA	Caspases-1 and -8	Acute myocardial infarction in mice	Cardioprotective	[53]
BH4	VDAC activity	Fulminant Fas-induced liver failure and acute myocardial infarction in mice	Cardioprotective	[66]
SOD1/CAT	Antioxidative enzymes	Myocardial infarction in rats	Protected in a combined fashion against IRI	[67]
haFGF	Brain neurons	Mouse model of Alzheimer’s disease	Neuroprotective (reduce amyloid protein deposits)	[82]
Neuroglobin	Cerebral neurons	Middle cerebral artery occlusion in mice	Reduction of infarct size	[91]
Hsp70	Chaperone activity	Transient focal cerebral ischemia in mice	Neuroprotective in stroke	[96]
NEMO	NF-κB pathway	Inflammatory bowel disease (IBD) in rats	Ameliorates TNBS-induced colitis	[97]
D-isomer of p53	Reactivation of p53	Peritoneal carcinomatosis in mice	Increase longevity of mice harboring lymphoma	[99]
PNP	PNP replacement therapy	Metabolic disorder in mice	Corrects gene deficiency	[108]

For a long time it was assumed that apoptosis was the only regulated form of cell death. Therefore, most successful applications of CPP-assisted delivery of proteins or peptides dealing with RCD have been reported in the context of apoptotic signaling pathways. Our own group discovered that incubation of lymphocytic Jurkat or BJAB cells with a recombinant Tat-FLIP_s_ fusion protein significantly inhibits Fas-induced activation of procaspase-8 and downstream caspases, preventing cells from undergoing apoptosis. Systemic application of this protein prolongs survival and reduces multiorgan failure due to otherwise lethal Fas-receptor-mediated apoptosis in vivo [52]. Given that in clinically relevant cell death, the intrinsic and the extrinsic apoptotic pathways often synergistically contribute to organ failure, we developed Tat-crmA, a fusion protein that is capable of blocking key caspases of both pro-apoptotic pathways which was demonstrated in a murine cardiac model. Therein, a single therapeutic application of Tat-crmA reduced infarction size by 40 % and preserved left ventricular function [53]. Protection against ischemic brain injury and neuronal apoptosis in mammals has also been reported with a Tat-Bcl-xL fusion protein which is transduced very efficiently into primary neurons [63]. Both Tat-Bcl-xL and Tat-crmA are effective even when they were administered after the completion of ischemia and proved that CPPs are able to cross the blood–brain barrier in an active manner ([63] and our own unpublished data).

A related approach showed that the conserved N-terminal homology domain (BH4) of Bcl-xL alone, after fusion to the protein transduction domain of HIV Tat, closes voltage-dependent anion channels (VDAC) and thus efficiently inhibits mitochondrial cell death [64]. The same group showed in a follow-up study that the application of Tat-BH4 inhibited X-ray induced apoptosis in the small intestine of mice, and suppressed Fas-induced fulminant hepatitis and heart failure after ischemia–reperfusion injury in isolated rat organs [65]. Unfortunately, we were not able to confirm these very interesting findings because we have never seen a vehicle as insoluble as the BH4 domain of Bcl-xL. The therapeutic potential of the CPP-conjugated BH4 domain was additionally indicated in a murine model of acute myocardial infarction which showed the significant cardioprotective properties of this peptide after a single bolus of intravenous injection [66]. With respect to the regulation of mitochondrial membrane permeability, this kind of regulated cell death in the model above is often connected globally with apoptosis, but we have shown previously that regulated necrosis may also result from a cyclophilin D-mediated mitochondrial permeability transition, revealing that the cellular context requires a balanced interplay between these two modes of cellular demise [40]. Furthermore, we were able to demonstrate in this mentioned study that combination therapies targeting distinct regulated necrosis pathways can be beneficial in the treatment of ischemic injury. In a similar case, it was shown that combined use of PEP-1-superoxide dismutase 1 (SOD1) and PEP-1-catalase (CAT) fusion proteins protects the myocardium from ischemia–reperfusion-induced injury in rats to a significantly higher extent as each single one CPP alone [67]. Of course, the chosen proteins SOD1 and CAT did not really affect distinct signaling pathways, but it is remarkable that two antioxidant enzymes cooperatively protected an organ against IRI.

Bcl-xL and the BH4 domain of Bcl-xL, respectively, are worthy of discussion in a further context of therapeutic CPP application. In a previous work, we were able to show that RIPK3-deficient mice, in contrast to C57BL/6 wild-type mice, were significantly protected from TNFα-induced and TNFα/zVAD-mediated hyperacute shock [68]. Engagement of TNF receptor 1 signals tended, depending on cellular background and milieu, towards caspase-dependent apoptosis or RIPK-dependent necroptosis. In this context, the group of Peter Vandenabeele detected, moreover, that pretreatment of wild-type mice with the RIPK1 kinase inhibitor necrostatin-1 provided a similar effect in this approach [69], a phenomenon which we cannot confirm, independently of the commonly used TNFα concentration [68]. Nevertheless, both approaches indicate that RIPK1/RIPK3-mediated cellular damage by necrosis drives mortality during TNFα-induced systemic inflammatory response syndrome (SIRS). Furthermore, the Vandenabeele group proved in this setting that RIPK3 deficiency also protected against cecal ligation and puncture, underscoring the clinical relevance of this protein kinase in sepsis. Nevertheless, it is remarkable that previous animal studies explicitly described that prevention of apoptosis and not necroptosis in animal models of *Escherichia coli*-induced sepsis improves survival. In such a study, Hotchkiss et al. proved an apoptotic signaling in this approach by application of a Tat-conjugated Bcl-xL fusion protein or Tat-BH4 peptide, respectively [70]. Therein, both CPPs markedly decreased lymphocyte apoptosis in an in vivo mouse model of sepsis which scrutinized the real mode of regulated cell death in sepsis. The role of caspases and their inhibitors in sepsis to cause and protect against apoptosis, inflammation, pyroptosis and necroptosis has been summarized in [71]. Doubtless, caspase inhibitors as well as caspase deficiency greatly improve the survival and overall disease outcome in sepsis models [72], but Cauwels et al. showed equally that co-administration of the pan-caspase inhibitor zVAD sensitized mice to TNFα induced SIRS [73]. The discrepancies in these reports regarding the involvement of apoptosis or necroptosis in a disease model indicate that it is necessary to update our understanding of regulated cell death processes, particularly given that signaling platforms like the ripoptosome can switch modes between apoptotic and necroptotic cell death [74].

Beside the transforming growth factor-β activated kinase-1 (TAK1 kinase), the adaptor MyD88 (myeloid differentiation primary response gene 88) is a suitable candidate which is able to mediate the decision to die by apoptosis or necroptosis [75, 76]. MyD88 has a pivotal role in Toll-like-receptor (TLR) and IL-1R signaling and is involved in mediating excessive inflammation. The inhibition of this unwanted response in acute and chronic inflammatory diseases mediated by MyD88 has significant therapeutic potential. MyD88 is composed of a death domain and a Toll/IL-1R domain connected by an intermediary domain (INT). Based on the fact that MyD88 lacking INT is not able to transmit signals to the downstream kinase IRAK4, the group of Roman Jerala fused a synthetic INT peptide consisting of 21 amino acids to different CPPs and demonstrated that the injection of these different constructs into mice challenged by an otherwise lethal dose of LPS significantly suppressed the production of IL-6 and TNFα and significantly improved the survival of the mice [77].

Oxidative stress, or rather the formation of reactive oxygen species (ROS), plays a significant role in effecting cellular pathogenesis which contributes to regulated cell death in many neurological diseases such as stroke, brain trauma, Parkinson’s diseases and Alzheimer’s diseases [78]. Like in sepsis, the proper multi-faceted signaling complexes contributing to this form of RCD are not unique and sometimes controversial. Nevertheless, literature indicates that necroptosis as well as apoptosis may be responsible for cellular dysfunctions leading to different modes of neurodegenerative diseases [45, 79]. These findings are also in line with a study which described that wild-type p53 can induce both apoptosis and ferroptosis upon ROS-induced stress [80]. How exactly p53 changes its function to elicit different forms of cell death is an important but so far unresolved question which remains to be elucidated. Nevertheless, by such applied research we will better understand the level of entangled molecular processes and pathways contributing to cell death subroutines within a complex etiopathology.

In an approach to ameliorate neurodegenerative diseases in patients, the group of Yadong Huang described in consecutive studies proteins consisting of human acidic fibroblast growth factor (haFGF) coupled to the Tat-domain of HIV [81–83]. Delivery of therapeutics into the brain is a major challenge because of the blood–brain and blood-cerebrospinal fluid barriers. Many traditional drugs cannot cross these barriers in appreciable concentrations, with less than 1 % of most drugs reaching the central nervous system, leading to a lack of marketable treatments for many neurological diseases such as neurodegenerative disorders, epilepsy, stroke and malignant brain tumors [84]. In a well- characterized mouse model of Alzheimer’s disease using the senescence-accelerated mouse prone 8 (SAMP8) mice, intranasally administrated CPP-conjugated acidic fibroblast growth factor fusions proteins retained the neuroprotective activities of the neurotrophin-like growth factor in the brain over a period of several weeks, and, therefore may be promising candidates for the development, differentiation and regeneration of brain neurons and consequently treatment of neurodegenerative diseases [82]. Above all, the fusion proteins significantly reduced β-amyloid protein deposits in the brain and thus protected the neurons from RCD. Accessory studies pointed out that the RIPK1/RIPK3 necrosome can form such functional amyloid signaling complexes which could be responsible for disease progression [85]. The combination of such reports highlights once more the concept of peptide-based strategies in the context of intervention in degenerative processes, especially with regard to the dramatic acceleration in the discovery of new RCD modes during the last decade [86].

Antigen-induced blockade of airway inflammation and hyperresponsiveness in mice were evaluated in an elegant study using a dominant negative Ras that was fused to the Tat protein transduction domain. At the cellular level, the CPP-carrying mutated Ras in this model inhibits the migration and infiltration of inflammatory cells, largely eosinophils and mononuclear cells as well as IL-4 and IL-5 production in the lung [87].

Additionally worth mentioning is an interesting study by Rothbard and colleagues which includes a category of molecules that normally do not require assistance for their uptake into cells. In general, small molecules which are used successfully for inhibition of different forms of RCD (e.g. necrostatin-1 or ferrostatin-1), or that operate as chemotherapeutic drugs (e.g. doxorubicin or cyclosporin A) are *per se* cell-permeable and therefore need no further vehicles for transduction into target cells. That is why the immunosuppressive cyclosporin A would be an ideal component for treatment of inflammatory skin disorders mediated by dermal T lymphocytes. Unfortunately, this drug is only locally active and ineffective topically because of poor penetration into the skin. To overcome this hurdle, the aforementioned group of Rothbard conjugated a heptamer of arginine to cyclosporine A and demonstrated in a compelling fashion that, in contrast to the pure cyclosporin A, this modified drug was efficiently transported into the epidermis and dermis of mouse and human skin and as a result inhibited cutaneous inflammation [88]. This approach established a novel strategy for enhancing the delivery of poorly absorbed drugs across tissue barriers. Since resistance to chemotherapeutic agents, including paclitaxel, methotrexate or glucocorticoids, is a strong predictor of poor outcome in cancer therapy, it was proposed that modulation of cell death regulators might represent a novel and promising strategy for counteracting drug resistance in these cells [89].

Former studies performed in mice support that Tat-conjugated proteins can be efficiently transduced into neurons and protect the brain from mild or moderate ischemic injury [90]. Mammalian neuroglobin (Ngb) has been suggested to be able to protect against brain hypoxic-ischemic injury. Unfortunately, such natural inhibitors which are produced in the brain in response to acute cerebral ischemia cannot be developed for treatment of stroke because these potentially therapeutic proteins do not cross the blood–brain barrier. By conducting middle cerebral artery occlusion (MCAO), Cai et al. proved that the concomitant application of a recombinant Tat-Ngb fusion protein in this model resulted in significantly (about one-third) less brain infarction volume, manifested in a better neurological outcome, if Tat-Ngb was injected intravenously 2 h before the reperfusion was initiated [91]. It is important to note that the protection observed was negligible when the fusion protein was applied at the onset of reperfusion. This last remark illustrates a general vulnerability of many potential drugs; their application may be too late when a severe incident has been diagnosed in the patient.

Unfortunately, stroke triggers a complex series of biochemical mechanisms and our knowledge of the molecular mechanisms of stroke pathophysiology is constantly advancing. In this context, some fundamental proteins like MAPKs (ERK, p38 and JNK) are involved in oxidative stress pathways and inflammation, while others like caspases, Bcl-2 family proteins, PARP-1, apoptosis-inducing factor (AIF), inhibitors of apoptosis proteins (IAPs), receptor interacting protein kinases (RIPKs) and heat-shock protein 70 (Hsp70) are involved in RCD pathways [92]. The damaging mechanisms of stroke may proceed through rapid nonspecific cell lysis (passive necrosis) or by active forms of cell demise (apoptosis or regulated necrosis), depending upon the severity and duration of the ischemic insult. Therefore, selecting promising targets for drug discovery from these various signaling cascades is challenging. The chaperone Hsp70 is a molecule that reduces ischemia/reperfusion injury in the brain and evidence is emerging that the activation of key players of RCD pathways often requires chaperones and co-chaperone complexes [93–95]. It was shown that treatment of transient focal cerebral ischemia in mice with a recombinant Tat-Hsp70 fusion protein resulted in a significantly smaller infarction size and in functional improvement compared with corresponding controls [96]. Such studies suggest that chaperones conjugated to CPP may represent an alternative class of neuroprotective therapeutics against stroke.

A further clue to the applicability of CPPs in the course of RCD processes resulted from a study dealing with the transcription factor NF-κB, a central regulator of the immune response. It has been known for a long time that compounds which directly inhibit the binding of NF-κB to DNA may block inflammation and the associated tissue damage. Briefly, NF-κB essential modulator (NEMO) is a component of the IKK complex which participates in the activation of the NF-κB pathway. It was shown that the colon-targeted cell-permeable NF-κB inhibitory peptide TALDWSWLQTE is active against experimental colitis in vivo and indicates the therapeutic targeting of NF-κB for treatment of inflammatory bowel disease (IBD). Tissue permeability and colon availability of this NEMO binding domain peptide was examined spectro-photometrically using FITC-labeled constructs [97].

Traditionally, therapeutics that restore genes inactivated during oncogenesis have been of superordinate interest in basic and translational science. It has long been speculated that direct reactivation of the endogenous tumor suppressor p53 in cancer cells will be therapeutically beneficial. So far, many different p53-derived peptides conjugated to various CPPs like penetratin or Tat have been demonstrated to restore the tumor suppressor function of p53 in cancer cells (reviewed in [98]). The group of Steven Dowdy demonstrated in 2004 that a retro-inverse version of the parental p53 peptide linked to the Tat-domain of HIV (named RI-Tatp53C) restored p53 function specifically in cancer cells but not in normal cells [99]. Using a peritoneal lymphoma model they demonstrated that treatment of mice with this transducible d-isomer of p53 resulted in significant increases in lifespan and the generation of disease-free animals. This approach appears valuable because it was shown previously that p53 inhibits cystine uptake and thereby sensitizes cells to ferroptosis, an oxidative, iron-dependent, non-apoptotic form of RCD [100]. Ferroptosis has recently been described and implicated in several pathological conditions including Huntington´s disease and kidney dysfunction [41, 101]. It is an intriguing idea that reactivation of the endogenous p53 in cancer cells using CPPs such as RI-Tatp53C could promote the selective removal of cancer cells through induction of ferroptosis.

However, unequivocal controls are most important for applying the technology of CPPs. Correspondingly, the interpretation of necrotizing pancreatitis in regard to RCD is somewhat disputed. Some works describe that RIPK3 deficiency in mice partially protects against cerulein-induced pancreatitis (CIP), indicating the involvement of necroptotic signaling pathways in the course of this disease [102, 103]. However, we have shown previously a deterioration of pancreatic damage in this model upon addition of the RIPK1 inhibitor Nec-1 and our recombinant Tat-crmA fusion protein, respectively [68]. Nevertheless, it is striking that pretreatment of rats with the penetratin domain alone (without any further cargo) which rapidly entered the cells of the pancreas, attenuated the severity of pancreatic inflammation monitored by different serum parameters of pancreatitis and associated oxidative stress [104].

In modern medicine, molecularly targeted imaging is gaining increasing significance. CPPs long ago became important tools for delivering and detection of fluorophores or luminescent nanocrystals such as quantum dots [105]. Furthermore, the grading of primary tumors and metastases is a fundamental process in cancer therapy. The extent, evaluation and accurate identification of metastases often requires surgical removal of all anatomically susceptible lymph nodes for pathological examination. Using advantages of ratiometric ACPPs, Savariar and coworkers recently established an impressive, novel method of real-time in vivo imaging of tumors [106]. The activatable peptides contain Cy5 as a far-red fluorescent donor and Cy7 as near-infrared fluorescent acceptor. Cy5 is quenched in favor of Cy7 re-emission until the intervening linker is cut by tumor-associated matrix metalloproteinases, which play crucial roles in cancer invasion and metastasis. The attack of the protease disrupts fluorescence resonance energy transfer (FRET), increases the Cy5/Cy7 emission ratio in a time-dependent manner and triggers tissue retention of the Cy5-labeled peptide. This ratiometric increase provides an accelerated and quantifiable metric to identify primary tumors and metastases in liver and lymph nodes with increased sensitivity and specificity. The application of CPPs in oncology represents a significant advance over existing non-ratiometric protease sensors and sentinel lymph node detection methods, which give no information about cancer invasion. Such developments illustrate the manifold range of CPP applications in medicine and prove that the technology of peptide delivery is continually undergoing refinement.

## Promises and pitfalls of cell-penetrating peptides

So far, a plethora of cargoes have been successfully delivered by CPPs or PTDs into a vast number of cell types and in over 25 clinical trials (Phase I and II) which have been performed predominantly using Tat-PTD, 8R and penetratin, evaluating this technique and its application as valuable and safe [107]. The current status of the most promising trials of CPP-mediated therapeutics is listed in Table 3. Transfer of highly promising basic scientific research into clinical applications arising for instance from a long-term study in mice (over 24 weeks) illustrated that a gene defect of the ubiquitous enzyme purine nucleoside phosphorylase (PNP) leading to severe T cell immunodeficiency and neuronal dysfunction can be corrected effectively by a recombinant Tat-PNP fusion protein [108]. Nevertheless, despite several promising lines of preclinical evidence, so far no PTD- or CPP-derived drug has passed the FDA or has reached the market. Of course, the efficiency of cytosolic access of these fusion protein conjugates is insufficient and their lack of specificity hinders widespread implementation in vivo and therefore remains a major hurdle for biomedical applications. So far, strategies to improve the specificity of CPP-conjugates have focused mostly on tumor targeting [109]. In the near future, cell-targeting peptides with an intrinsic cell-penetrating activity like so-called bioportides might expand the repertoire of strategies available to increase the selectivity of this new class of biopharmaceuticals [110]. So far, several notable cell death relevant constructs have been considered for clinical development. In detail: delcasertib (KAI-9803), a Tat-coupled PKCδ inhibitor which did not show a significant decrease in heart tissue damage from artery-opening being in a Phase II clinical trial, although the drug reduced cardiac damage in a rat model of acute myocardial infarction [111]; and XG-102, a Tat-coupled c-Jun N-terminal kinase (JNK) inhibitor which reduces myocardial ischemia–reperfusion injury and infarction size in rats [112] is currently in an ongoing Phase III clinical trial dealing, surprisingly, with the reduction of intraocular inflammation post-cataract surgery [113].

**Table 3:** List of selected currently ongoing clinical trials employing CPP-conjugates

Company	CPP-conjugate	Label	Indication	Outcome	References
CellGate, Inc.	R7-cyclosporine A	PsorBan^®^	Topical treatment of psoriasis	Phase II clinical trials terminated	[88]
Revance Therapeutics, Inc.	Tat-Botulinum toxin	RT-001	Topical treatment of facial wrinkles	Currently in a Phase III clinical development program	[129]
Capstone Therapeutics	PTD4-Hsp20	AZX-100	Prevention of dermal/keloid scarring	Phase II completed in 2012	[130]
Amgen	Tat-PKCδ inhibitor	KAI-9803	Acute myocardial infarction	Phase II completed in 2011	[131]
Amgen	Tat-PKCε inhibitor	KAI-1678	Neuropathic pain	Phase II completed in 2011: not efficacious	[132]
KAI Pharmaceuticals	Tat-PKCε activator	KAI-1455	Ischemic injury	Phase I initiated	[133]
Auris Medical (Xigen)	Tat-JNK inhibitor	XG-102	Inflammatory bowel disease	Phase I completed in 2012 (Xigen initiates enrolment in a phase III trial for ocular inflammation)	[113]
Avi Biopharma	PPMO^a^	AVI-5038	Duchenne muscular dystrophy	Currently in preclinical development	[134]
National AIDS Center, Rome (Italy) and Novartis	Tat-V2-deleted Env proteins	ISS T-002	HIV vaccine (therapeutic immunization)	Entered Phase II	[135]
Traversa Therapeutics/Sanofi-Aventis	multiple PTD siRNAs	PTD-DRBD^b^	Degradation of target mRNA	Preclinical studies	[136]
Diatos and Drais Pharmaceuticals	Vectocell^®^-SN38	DTS-108	Cancer treatment	Phase I	[137]
NoNO Inc.	Tat-NR2B9c	NA-1	Ischemic brain damage	Phase III	[114]

^a^Penetrating phosphodiamidate morpholino oligomer for skipping dystrophin gene

^b^Double-stranded RNA binding domain

At this time, the most promising candidate to become approved by the FDA may be NA-1 (Tat-NR2B9c), a peptide disrupting the *N*-methyl-d-aspartate receptor-postsynaptic density protein-95 interaction, a pro-death signaling pathway. This drug attenuates ischemic damage in the acute phase after stroke without affecting cerebral blood flow in the ischemic core or penumbra. A Phase II clinical trial proved the safety and efficacy of NA-1 in patients with iatrogenic stroke after endovascular aneurysm repair [114]. Therefore, this trial has the potential to revolutionize the treatment of a wide spectrum of human diseases using therapeutically effective CPPs.

Reports illustrating noticeable off-target effects of CPPs *per se* are interesting for scientists but detrimental for a contemplated clinical evaluation. In such a manner, cationic CPPs, like the Tat-domain, sometimes evoke side-effects by remodeling the plasma membrane. Transduction of Tat-GFP fusion proteins causes membrane phospholipid rearrangement as evidenced by detection of phosphatidylserine (PS) on the outer surface of the cell membrane. Remarkably, neither apoptosis nor necrosis is induced in these cells after exposure to Tat-GFP [115]. This observation was extended by another approach, demonstrating that CPPs are occasionally able to induce changes in the lipid composition of the plasma membrane. The mechanism involves the induction of acid sphingomyelinase (ASMase), which converts sphingomyelin to ceramide at the outer leaflet of the plasma membrane. The involvement of ASMase in CPP uptake was confirmed by a pharmacological inhibition of ASMase by imipramine and a subsequent rescue of uptake through external addition of sphingomyelinase, and using ASMase-deficient cells [116]. These findings could have important implications for cancer therapy because ceramide is the central molecule of sphingolipid metabolism and a lipid with important second messenger functions which mediates anti-proliferative responses and induction of apoptosis as well as necroptosis [117].

## Concluding remarks and perspectives

Despite extensive progress in recent years resulting in promising lines of preclinical evidence, more work is still needed and desirable for biomedical applications of therapeutically exploitable proteins. The low cell, tissue and organ selectivity of first generation CPPs in mammals in combination with the low protein transduction efficiency is a tremendous drawback of this technology and the therapeutic application of these carriers. In addition, the in vivo utility of CPPs may be compromised by the general capacity of polycationic peptides to activate mast cell secretion [118]. Moreover, the route of administration is a meaningful concern. Oral delivery of pharmaceutical peptides or proteins is still a major challenge for pharmaceutical industries. Negligible permeability of these drugs across the intestinal barrier and low pH conditions of the gastrointestinal tract reduce the delivery of intact agents to the target and add to the short half-life and low bioavailability of CPPs. In this regard, it is worth noting that Xentry [119], as already mentioned, represents a new class of cell-penetrating peptides that is not able to invade resting blood cells, which offers a therapeutic advantage when the drug must be administrated intravenously. Furthermore, nano-carrier-based delivery represents an appropriate choice to significantly improve uptake of CPPs and protect cargo from proteolytic degradation with the intention of increasing the retention time of these molecules in the body, leading to a reduction of treatment cost [120]. d-amino acids which are less susceptible to protease activity than the biologically used l-form could be considered an alternative for preparation of CPPs to increase serum stability. Such, it was revealed that d-isomeric Xentry was stable in serum for 4 h, much longer than the l-isomer which became inactive within 1 h [11].

The challenge remains to find a compromise between avoiding the premature degradation of the cargo and obtaining a sufficiently effective drug release rate from the CPP-conjugate after internalization. Activatable cell-penetrating peptides (ACPPs) have an enhanced ability to penetrate their target cells within primary tumors or metastases compared to conventional CPPs due their cleavage by disease-associated matrix metalloproteinases and could eliminate background activity in healthy cells in such a manner [27]. Potential side-effects from therapeutic molecules such as toxicity and undesired immune responses must also be considered.

Without any doubt, the current door-to-balloon-time appears to counteract the successful and effective prevention of RCD in myocardial infarction. In this regard, it is notable that organ transplantation is unavoidably associated with transient tissue hypoxia, which may cause ischemia–reperfusion injury (IRI), inflammation and rejection. Our own studies demonstrated that kidney ischemia–reperfusion is a form of acute kidney injury resulting in a cascade of cellular events prompting rapid cellular damage and suppression of kidney function. In this context, we have shown for the first time that necroptosis is a key element of IRI in the kidney and therefore will likely emerge as a promising target in solid organ transplantation [121]. This is due to the fact that clinically feasible interference to attenuate organ injury may only be possible in situations in which the reperfusion damage or the mode of regulated cell death can be anticipated, such as cardiac surgery and solid organ transplantation. It is conceivable that CPPs could minimize the loss of functional parenchymal cells in the graft and/or prevent release of cell death-associated molecular patterns (CDAMPs) which trigger innate and promote adaptive immune responses and activate rejection pathways [122]. At present, the applicability of blocking necroptosis in transplantation is obvious, but has not been extensively tested. Discovery and synthesis of CPPs, readily penetrating cell membranes and concentrating primarily in mitochondria, may give new therapeutic perspectives. CPPs are now clearly established as a meaningful advance in the field of drug delivery in living organisms, but time will tell if this technology is also therapeutically useable to reset pathologic signaling networks in regulated cell death processes like transplantation.
